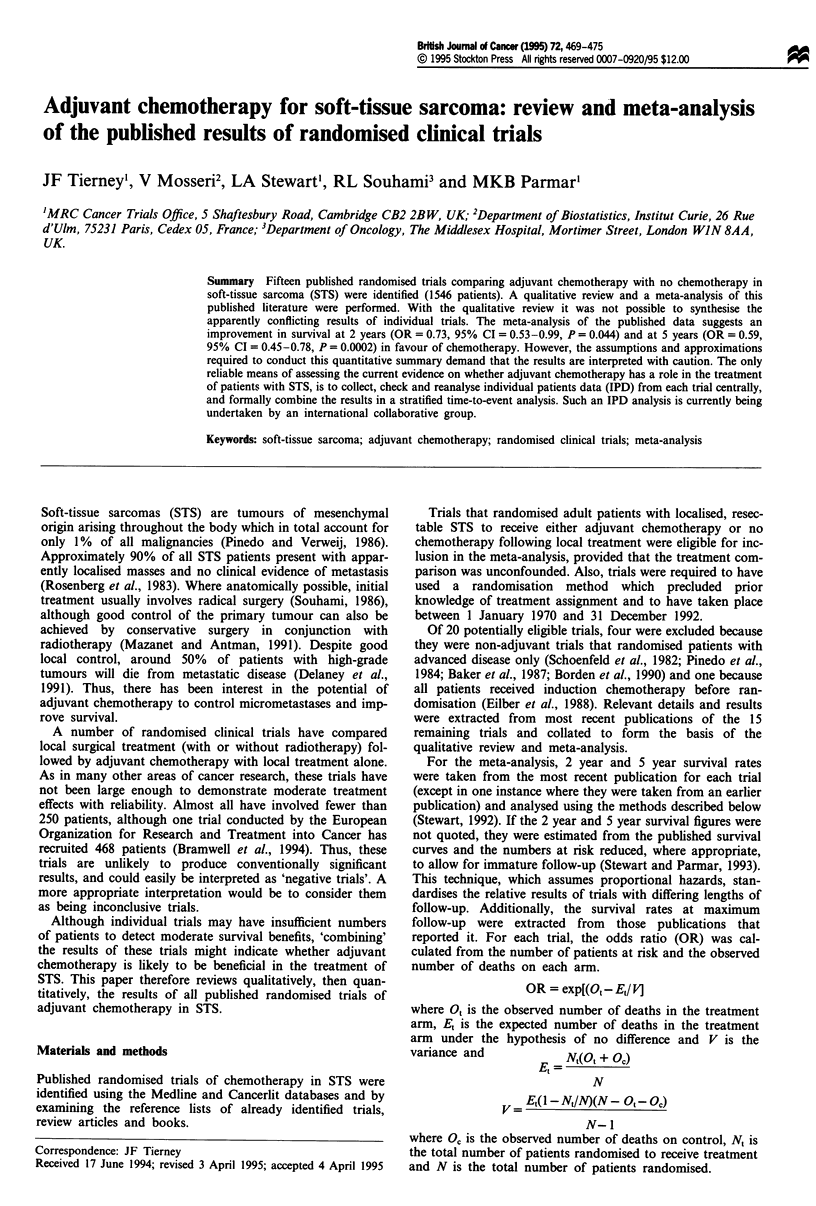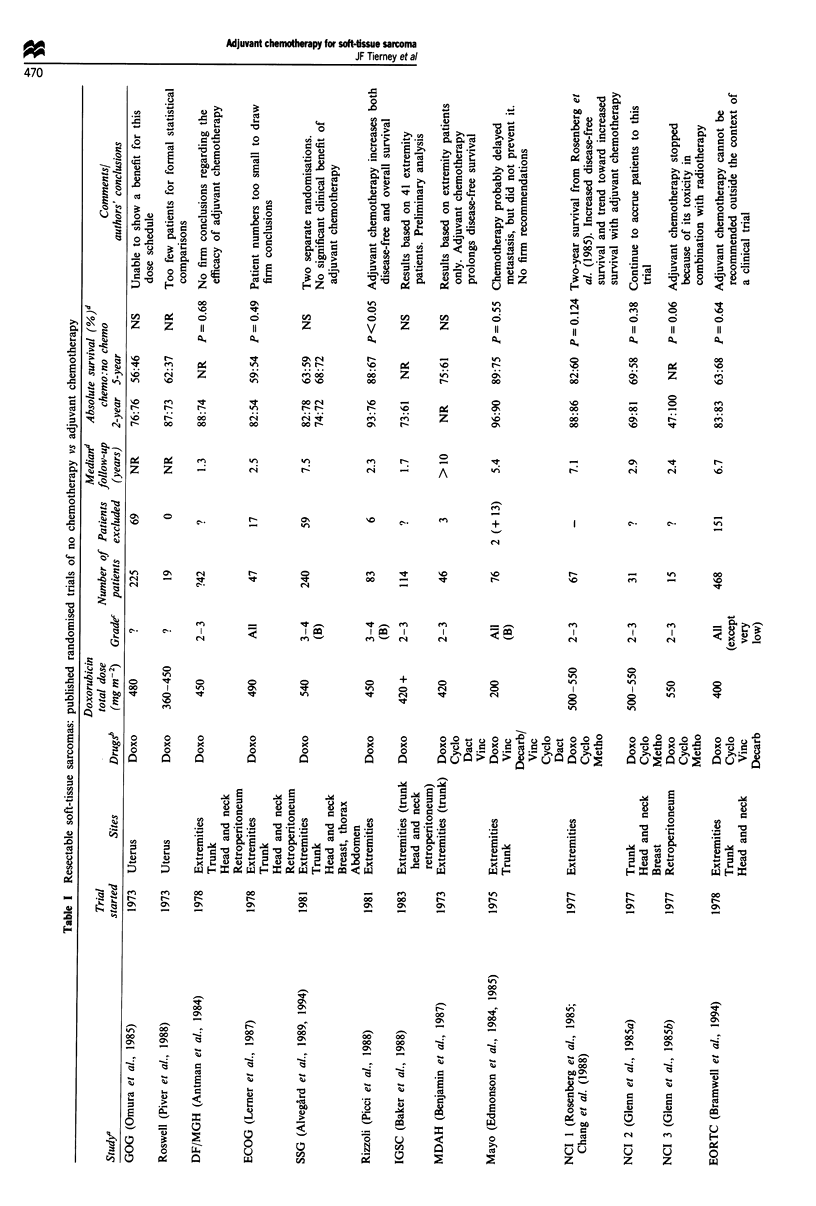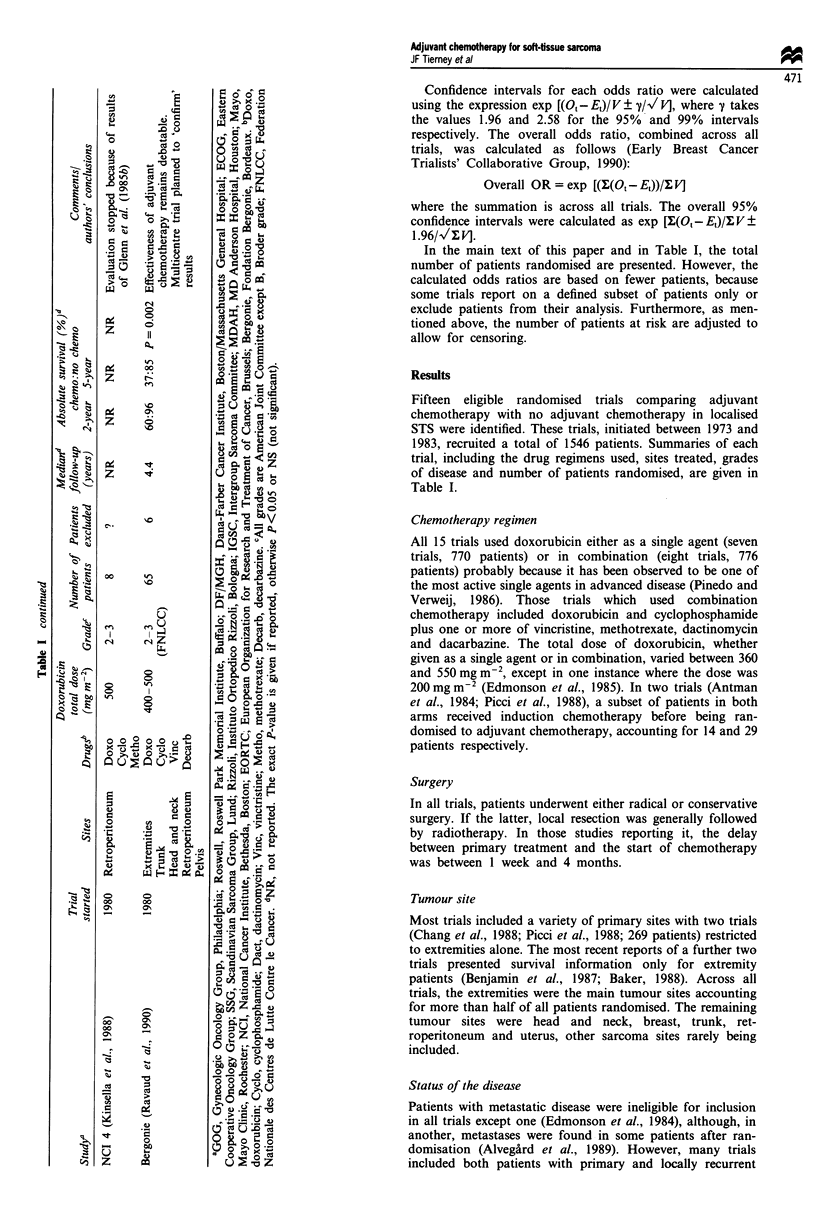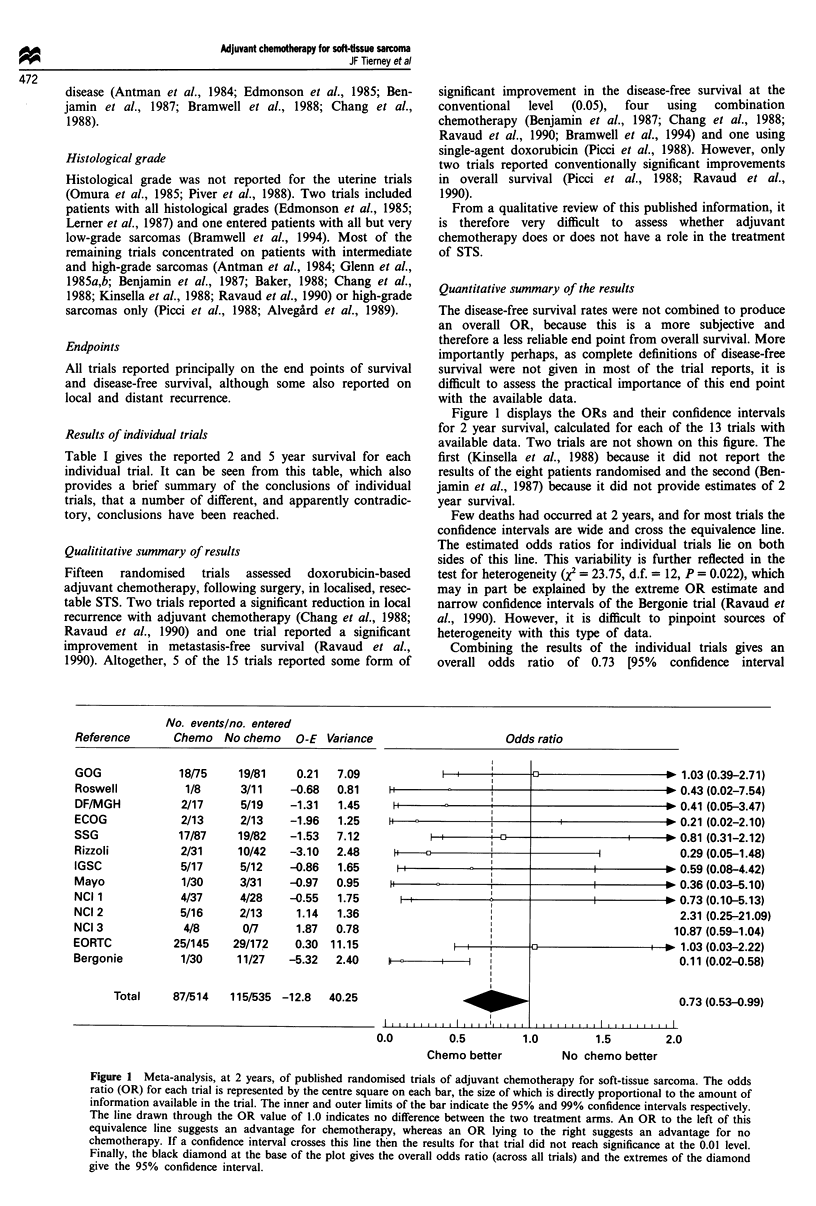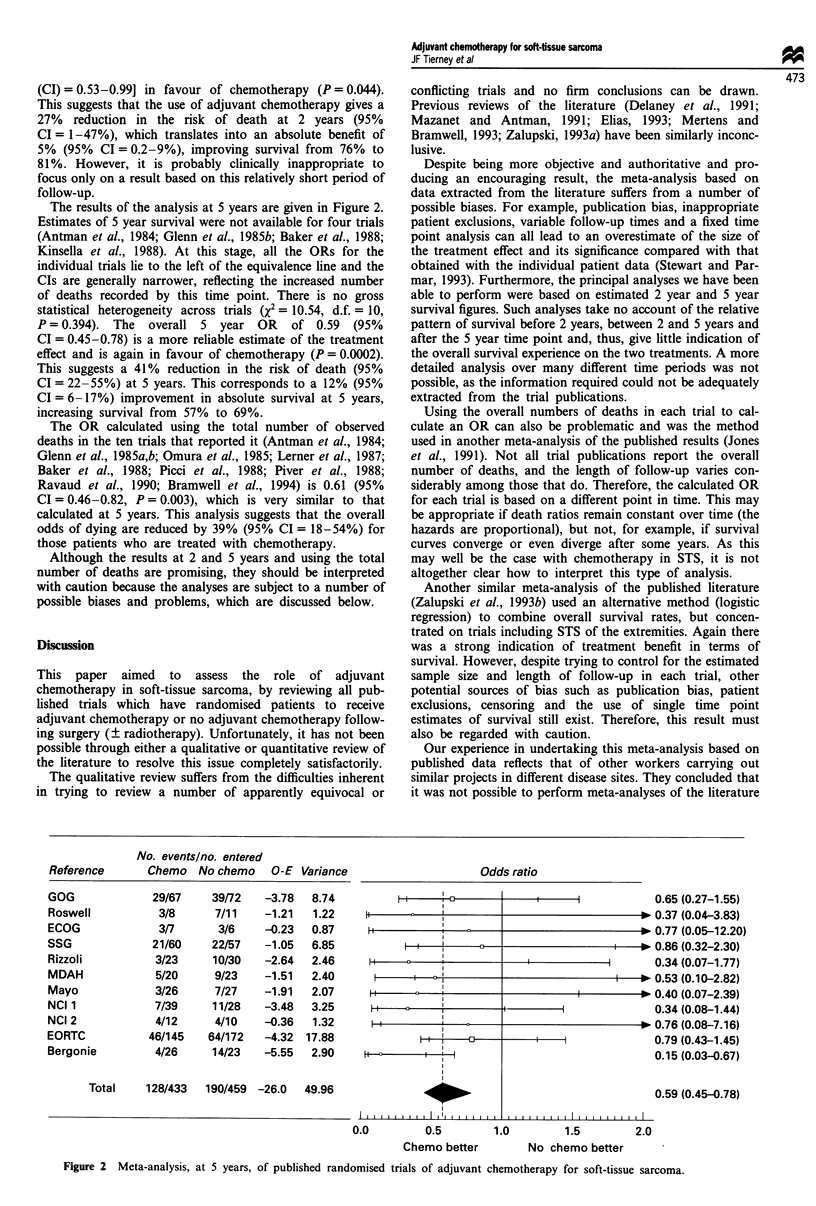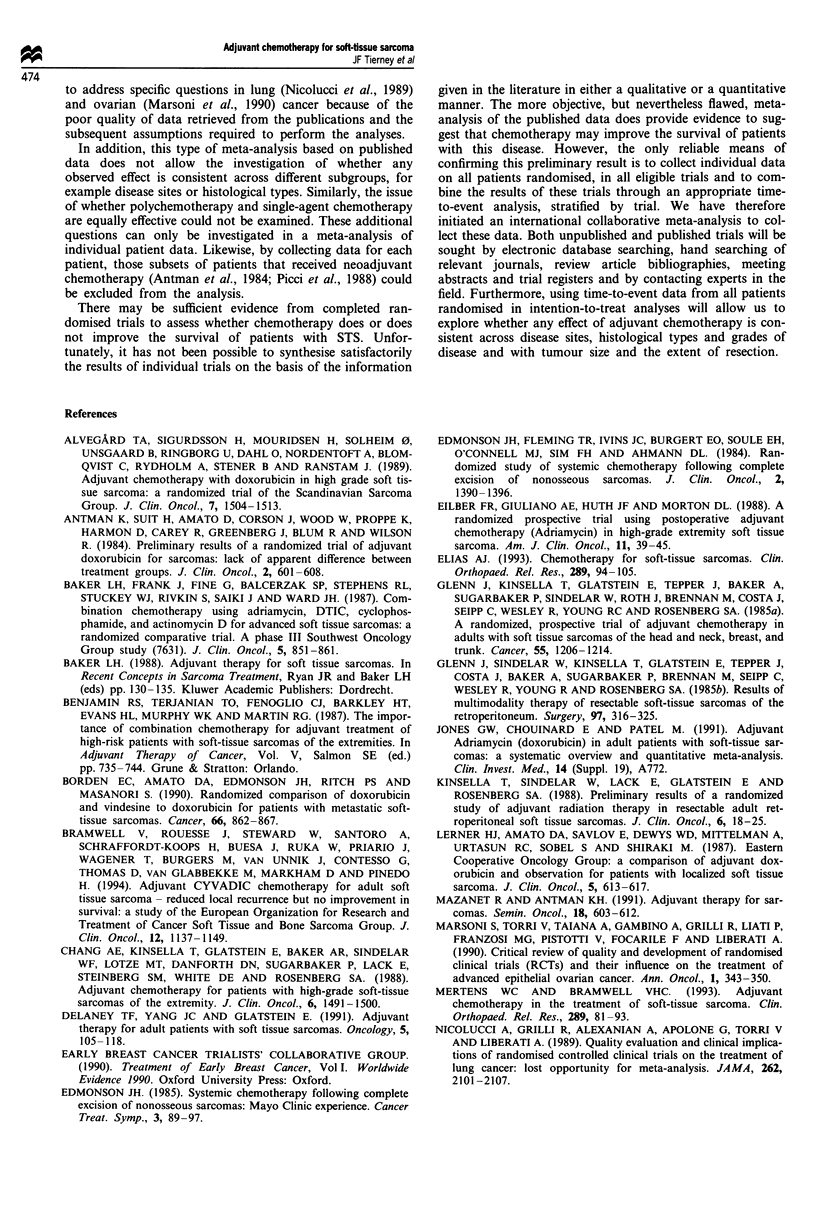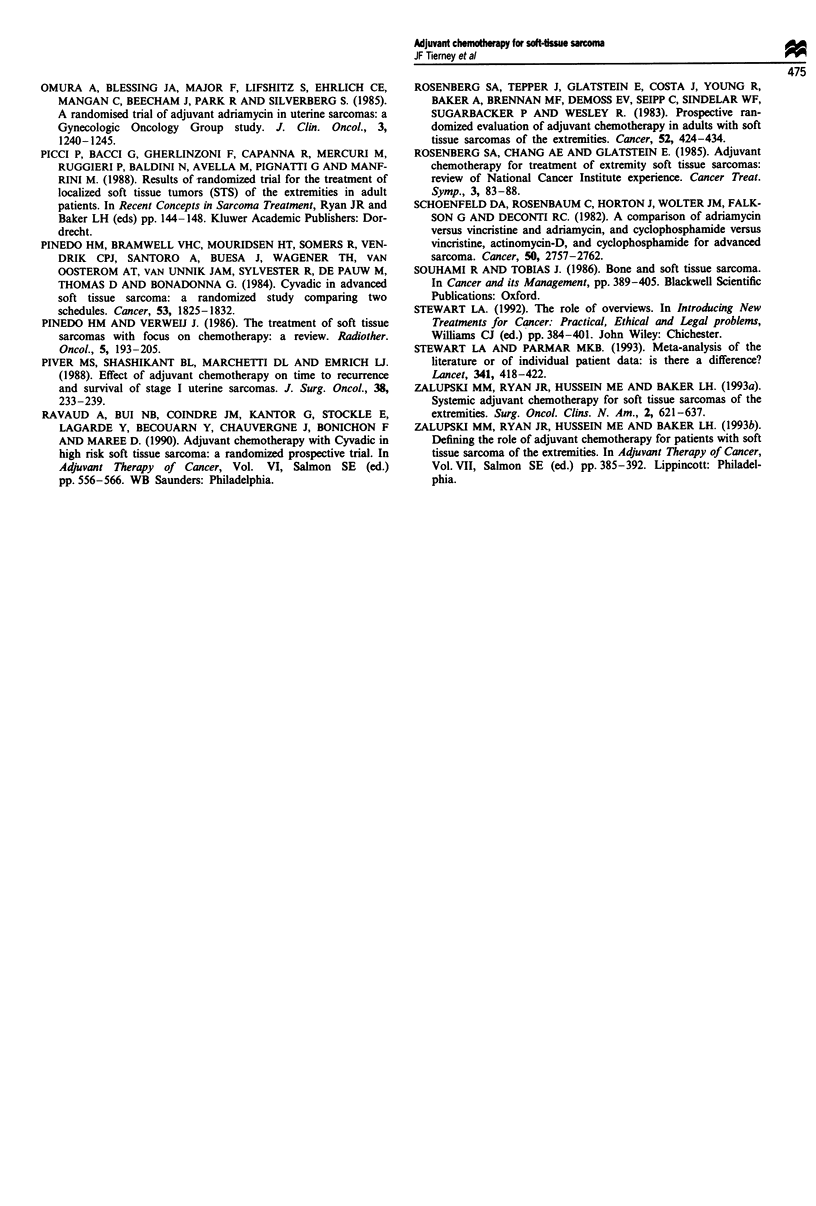# Adjuvant chemotherapy for soft-tissue sarcoma: review and meta-analysis of the published results of randomised clinical trials.

**DOI:** 10.1038/bjc.1995.357

**Published:** 1995-08

**Authors:** J. F. Tierney, V. Mosseri, L. A. Stewart, R. L. Souhami, M. K. Parmar

**Affiliations:** MRC Cancer Trials Office, Cambridge, UK.

## Abstract

Fifteen published randomised trials comparing adjuvant chemotherapy with no chemotherapy in soft-tissue sarcoma (STS) were identified (1546 patients). A qualitative review and a meta-analysis of this published literature were performed. With the qualitative review it was not possible to synthesise the apparently conflicting results of individual trials. The meta-analysis of the published data suggests an improvement in survival at 2 years (OR = 0.73, 95% CI = 0.53-0.99, P = 0.044) and at 5 years (OR = 0.59, 95% CI = 0.45-0.78, P = 0.0002) in favour of chemotherapy. However, the assumptions and approximations required to conduct this quantitative summary demand that the results are interpreted with caution. The only reliable means of assessing the current evidence on whether adjuvant chemotherapy has a role in the treatment of patients with STS, is to collect, check and reanalyse individual patients data (IPD) from each trial centrally, and formally combine the results in a stratified time-to-event analysis. Such an IPD analysis is currently being undertaken by an international collaborative group.


British Journal of Cancer (1995) 72, 469–475
© 1995 Stockton Press All rights reserved 0007-0920/95 $12.00

### Adjuvant chemotherapy for soft-tissue sarcoma: review and meta-analysis of the published results of randomised clinical trials

JF Tierney[1], V Mosseri[2], LA Stewart[1], RL Souhami[3] and MKB Parmar[1]

[1]*MRC Cancer Trials Office, 5 Shaftesbury Road, Cambridge CB2 2BW, UK;* [2]*Department of Biostatistics, Institut Curie, 26 Rue d'Ulm, 75231 Paris, Cedex 05, France;* [3]*Department of Oncology, The Middlesex Hospital, Mortimer Street, London W1N 8AA, UK.*

**Summary** Fifteen published randomised trials comparing adjuvant chemotherapy with no chemotherapy in soft-tissue sarcoma (STS) were identified (1546 patients). A qualitative review and a meta-analysis of this published literature were performed. With the qualitative review it was not possible to synthesise the apparently conflicting results of individual trials. The meta-analysis of the published data suggests an improvement in survival at 2 years (OR = 0.73, 95% CI = 0.53–0.99, $P = 0.044$) and at 5 years (OR = 0.59, 95% CI = 0.45–0.78, $P = 0.0002$) in favour of chemotherapy. However, the assumptions and approximations required to conduct this quantitative summary demand that the results are interpreted with caution. The only reliable means of assessing the current evidence on whether adjuvant chemotherapy has a role in the treatment of patients with STS, is to collect, check and reanalyse individual patients data (IPD) from each trial centrally, and formally combine the results in a stratified time-to-event analysis. Such an IPD analysis is currently being undertaken by an international collaborative group.

**Keywords:** soft-tissue sarcoma; adjuvant chemotherapy; randomised clinical trials; meta-analysis

Soft-tissue sarcomas (STS) are tumours of mesenchymal origin arising throughout the body which in total account for only 1% of all malignancies (Pinedo and Verweij, 1986). Approximately 90% of all STS patients present with apparently localised masses and no clinical evidence of metastasis (Rosenberg *et al.*, 1983). Where anatomically possible, initial treatment usually involves radical surgery (Souhami, 1986), although good control of the primary tumour can also be achieved by conservative surgery in conjunction with radiotherapy (Mazanet and Antman, 1991). Despite good local control, around 50% of patients with high-grade tumours will die from metastatic disease (Delaney *et al.*, 1991). Thus, there has been interest in the potential of adjuvant chemotherapy to control micrometastases and improve survival.

A number of randomised clinical trials have compared local surgical treatment (with or without radiotherapy) followed by adjuvant chemotherapy with local treatment alone. As in many other areas of cancer research, these trials have not been large enough to demonstrate moderate treatment effects with reliability. Almost all have involved fewer than 250 patients, although one trial conducted by the European Organization for Research and Treatment into Cancer has recruited 468 patients (Bramwell *et al.*, 1994). Thus, these trials are unlikely to produce conventionally significant results, and could easily be interpreted as 'negative trials'. A more appropriate interpretation would be to consider them as being inconclusive trials.

Although individual trials may have insufficient numbers of patients to detect moderate survival benefits, 'combining' the results of these trials might indicate whether adjuvant chemotherapy is likely to be beneficial in the treatment of STS. This paper therefore reviews qualitatively, then quantitatively, the results of all published randomised trials of adjuvant chemotherapy in STS.

### Materials and methods

Published randomised trials of chemotherapy in STS were identified using the Medline and Cancerlit databases and by examining the reference lists of already identified trials, review articles and books.

Correspondence: JF Tierney
Received 17 June 1994; revised 3 April 1995; accepted 4 April 1995

Trials that randomised adult patients with localised, resectable STS to receive either adjuvant chemotherapy or no chemotherapy following local treatment were eligible for inclusion in the meta-analysis, provided that the treatment comparison was unconfounded. Also, trials were required to have used a randomisation method which precluded prior knowledge of treatment assignment and to have taken place between 1 January 1970 and 31 December 1992.

Of 20 potentially eligible trials, four were excluded because they were non-adjuvant trials that randomised patients with advanced disease only (Schoenfeld *et al.*, 1982; Pinedo *et al.*, 1984; Baker *et al.*, 1987; Borden *et al.*, 1990) and one because all patients received induction chemotherapy before randomisation (Eilber *et al.*, 1988). Relevant details and results were extracted from most recent publications of the 15 remaining trials and collated to form the basis of the qualitative review and meta-analysis.

For the meta-analysis, 2 year and 5 year survival rates were taken from the most recent publication for each trial (except in one instance where they were taken from an earlier publication) and analysed using the methods described below (Stewart, 1992). If the 2 year and 5 year survival figures were not quoted, they were estimated from the published survival curves and the numbers at risk reduced, where appropriate, to allow for immature follow-up (Stewart and Parmar, 1993). This technique, which assumes proportional hazards, standardises the relative results of trials with differing lengths of follow-up. Additionally, the survival rates at maximum follow-up were extracted from those publications that reported it. For each trial, the odds ratio (OR) was calculated from the number of patients at risk and the observed number of deaths on each arm.

$$\mathrm{OR} = \exp[(O_t - E_t/V]$$

where $O_t$ is the observed number of deaths in the treatment arm, $E_t$ is the expected number of deaths in the treatment arm under the hypothesis of no difference and $V$ is the variance and

$$E_t = \frac{N_t(O_t + O_c)}{N}$$

$$V = \frac{E_t(1 - N_t/N)(N - O_t - O_c)}{N-1}$$

where $O_c$ is the observed number of deaths on control, $N_t$ is the total number of patients randomised to receive treatment and $N$ is the total number of patients randomised.

**Table I** Resectable soft-tissue sarcomas: published randomised trials of no chemotherapy *vs* adjuvant chemotherapy

Confidence intervals for each odds ratio were calculated using the expression exp $[(O_t - E_t)/V \pm \gamma/\sqrt{V}]$, where $\gamma$ takes the values 1.96 and 2.58 for the 95% and 99% intervals respectively. The overall odds ratio, combined across all trials, was calculated as follows (Early Breast Cancer Trialists' Collaborative Group, 1990):

$$\text{Overall OR} = \exp\ [(\Sigma(O_t - E_t))/\Sigma V]$$

where the summation is across all trials. The overall 95% confidence intervals were calculated as exp $[\Sigma(O_t - E_t)/\Sigma V \pm 1.96/\sqrt{\Sigma V}]$.

In the main text of this paper and in Table I, the total number of patients randomised are presented. However, the calculated odds ratios are based on fewer patients, because some trials report on a defined subset of patients only or exclude patients from their analysis. Furthermore, as mentioned above, the number of patients at risk are adjusted to allow for censoring.

### Results

Fifteen eligible randomised trials comparing adjuvant chemotherapy with no adjuvant chemotherapy in localised STS were identified. These trials, initiated between 1973 and 1983, recruited a total of 1546 patients. Summaries of each trial, including the drug regimens used, sites treated, grades of disease and number of patients randomised, are given in Table I.

### *Chemotherapy regimen*

All 15 trials used doxorubicin either as a single agent (seven trials, 770 patients) or in combination (eight trials, 776 patients) probably because it has been observed to be one of the most active single agents in advanced disease (Pinedo and Verweij, 1986). Those trials which used combination chemotherapy included doxorubicin and cyclophosphamide plus one or more of vincristine, methotrexate, dactinomycin and dacarbazine. The total dose of doxorubicin, whether given as a single agent or in combination, varied between 360 and 550 $mg\ m^{-2}$, except in one instance where the dose was 200 $mg\ m^{-2}$ (Edmonson *et al*., 1985). In two trials (Antman *et al*., 1984; Picci *et al*., 1988), a subset of patients in both arms received induction chemotherapy before being randomised to adjuvant chemotherapy, accounting for 14 and 29 patients respectively.

### *Surgery*

In all trials, patients underwent either radical or conservative surgery. If the latter, local resection was generally followed by radiotherapy. In those studies reporting it, the delay between primary treatment and the start of chemotherapy was between 1 week and 4 months.

### *Tumour site*

Most trials included a variety of primary sites with two trials (Chang *et al*., 1988; Picci *et al*., 1988; 269 patients) restricted to extremities alone. The most recent reports of a further two trials presented survival information only for extremity patients (Benjamin *et al*., 1987; Baker, 1988). Across all trials, the extremities were the main tumour sites accounting for more than half of all patients randomised. The remaining tumour sites were head and neck, breast, trunk, retroperitoneum and uterus, other sarcoma sites rarely being included.

### *Status of the disease*

Patients with metastatic disease were ineligible for inclusion in all trials except one (Edmonson *et al*., 1984), although, in another, metastases were found in some patients after randomisation (Alvegård *et al*., 1989). However, many trials included both patients with primary and locally recurrent disease (Antman et al., 1984; Edmonson et al., 1985; Benjamin et al., 1987; Bramwell et al., 1988; Chang et al., 1988).

### Histological grade

Histological grade was not reported for the uterine trials (Omura et al., 1985; Piver et al., 1988). Two trials included patients with all histological grades (Edmonson et al., 1985; Lerner et al., 1987) and one entered patients with all but very low-grade sarcomas (Bramwell et al., 1994). Most of the remaining trials concentrated on patients with intermediate and high-grade sarcomas (Antman et al., 1984; Glenn et al., 1985a,b; Benjamin et al., 1987; Baker, 1988; Chang et al., 1988; Kinsella et al., 1988; Ravaud et al., 1990) or high-grade sarcomas only (Picci et al., 1988; Alvegård et al., 1989).

### Endpoints

All trials reported principally on the end points of survival and disease-free survival, although some also reported on local and distant recurrence.

### Results of individual trials

Table I gives the reported 2 and 5 year survival for each individual trial. It can be seen from this table, which also provides a brief summary of the conclusions of individual trials, that a number of different, and apparently contradictory, conclusions have been reached.

### Qualititative summary of results

Fifteen randomised trials assessed doxorubicin-based adjuvant chemotherapy, following surgery, in localised, resectable STS. Two trials reported a significant reduction in local recurrence with adjuvant chemotherapy (Chang et al., 1988; Ravaud et al., 1990) and one trial reported a significant improvement in metastasis-free survival (Ravaud et al., 1990). Altogether, 5 of the 15 trials reported some form of significant improvement in the disease-free survival at the conventional level (0.05), four using combination chemotherapy (Benjamin et al., 1987; Chang et al., 1988; Ravaud et al., 1990; Bramwell et al., 1994) and one using single-agent doxorubicin (Picci et al., 1988). However, only two trials reported conventionally significant improvements in overall survival (Picci et al., 1988; Ravaud et al., 1990).

From a qualitative review of this published information, it is therefore very difficult to assess whether adjuvant chemotherapy does or does not have a role in the treatment of STS.

### Quantitative summary of the results

The disease-free survival rates were not combined to produce an overall OR, because this is a more subjective and therefore a less reliable end point from overall survival. More importantly perhaps, as complete definitions of disease-free survival were not given in most of the trial reports, it is difficult to assess the practical importance of this end point with the available data.

Figure 1 displays the ORs and their confidence intervals for 2 year survival, calculated for each of the 13 trials with available data. Two trials are not shown on this figure. The first (Kinsella et al., 1988) because it did not report the results of the eight patients randomised and the second (Benjamin et al., 1987) because it did not provide estimates of 2 year survival.

Few deaths had occurred at 2 years, and for most trials the confidence intervals are wide and cross the equivalence line. The estimated odds ratios for individual trials lie on both sides of this line. This variability is further reflected in the test for heterogeneity ($\chi^2 = 23.75$, d.f. = 12, $P = 0.022$), which may in part be explained by the extreme OR estimate and narrow confidence intervals of the Bergonie trial (Ravaud et al., 1990). However, it is difficult to pinpoint sources of heterogeneity with this type of data.

Combining the results of the individual trials gives an overall odds ratio of 0.73 [95% confidence interval

| Reference | No. events/no. entered Chemo | No chemo | O-E | Variance | Odds ratio |
|---|---|---|---|---|---|
| GOG | 18/75 | 19/81 | 0.21 | 7.09 | 1.03 (0.39–2.71) |
| Roswell | 1/8 | 3/11 | –0.68 | 0.81 | 0.43 (0.02–7.54) |
| DF/MGH | 2/17 | 5/19 | –1.31 | 1.45 | 0.41 (0.05–3.47) |
| ECOG | 2/13 | 2/13 | –1.96 | 1.25 | 0.21 (0.02–2.10) |
| SSG | 17/87 | 19/82 | –1.53 | 7.12 | 0.81 (0.31–2.12) |
| Rizzoli | 2/31 | 10/42 | –3.10 | 2.48 | 0.29 (0.05–1.48) |
| IGSC | 5/17 | 5/12 | –0.86 | 1.65 | 0.59 (0.08–4.42) |
| Mayo | 1/30 | 3/31 | –0.97 | 0.95 | 0.36 (0.03–5.10) |
| NCI 1 | 4/37 | 4/28 | –0.55 | 1.75 | 0.73 (0.10–5.13) |
| NCI 2 | 5/16 | 2/13 | 1.14 | 1.36 | 2.31 (0.25–21.09) |
| NCI 3 | 4/8 | 0/7 | 1.87 | 0.78 | 10.87 (0.59–1.04) |
| EORTC | 25/145 | 29/172 | 0.30 | 11.15 | 1.03 (0.03–2.22) |
| Bergonie | 1/30 | 11/27 | –5.32 | 2.40 | 0.11 (0.02–0.58) |
| Total | 87/514 | 115/535 | –12.8 | 40.25 | 0.73 (0.53–0.99) |

**Figure 1** Meta-analysis, at 2 years, of published randomised trials of adjuvant chemotherapy for soft-tissue sarcoma. The odds ratio (OR) for each trial is represented by the centre square on each bar, the size of which is directly proportional to the amount of information available in the trial. The inner and outer limits of the bar indicate the 95% and 99% confidence intervals respectively. The line drawn through the OR value of 1.0 indicates no difference between the two treatment arms. An OR to the left of this equivalence line suggests an advantage for chemotherapy, whereas an OR lying to the right suggests an advantage for no chemotherapy. If a confidence interval crosses this line then the results for that trial did not reach significance at the 0.01 level. Finally, the black diamond at the base of the plot gives the overall odds ratio (across all trials) and the extremes of the diamond give the 95% confidence interval.

(CI) = 0.53–0.99] in favour of chemotherapy ($P = 0.044$). This suggests that the use of adjuvant chemotherapy gives a 27% reduction in the risk of death at 2 years (95% CI = 1–47%), which translates into an absolute benefit of 5% (95% CI = 0.2–9%), improving survival from 76% to 81%. However, it is probably clinically inappropriate to focus only on a result based on this relatively short period of follow-up.

The results of the analysis at 5 years are given in Figure 2. Estimates of 5 year survival were not available for four trials (Antman *et al.*, 1984; Glenn *et al.*, 1985*b*; Baker *et al.*, 1988; Kinsella *et al.*, 1988). At this stage, all the ORs for the individual trials lie to the left of the equivalence line and the CIs are generally narrower, reflecting the increased number of deaths recorded by this time point. There is no gross statistical heterogeneity across trials ($\chi^2 = 10.54$, d.f. = 10, $P = 0.394$). The overall 5 year OR of 0.59 (95% CI = 0.45–0.78) is a more reliable estimate of the treatment effect and is again in favour of chemotherapy ($P = 0.0002$). This suggests a 41% reduction in the risk of death (95% CI = 22–55%) at 5 years. This corresponds to a 12% (95% CI = 6–17%) improvement in absolute survival at 5 years, increasing survival from 57% to 69%.

The OR calculated using the total number of observed deaths in the ten trials that reported it (Antman *et al.*, 1984; Glenn *et al.*, 1985*a,b*; Omura *et al.*, 1985; Lerner *et al.*, 1987; Baker *et al.*, 1988; Picci *et al.*, 1988; Piver *et al.*, 1988; Ravaud *et al.*, 1990; Bramwell *et al.*, 1994) is 0.61 (95% CI = 0.46–0.82, $P = 0.003$), which is very similar to that calculated at 5 years. This analysis suggests that the overall odds of dying are reduced by 39% (95% CI = 18–54%) for those patients who are treated with chemotherapy.

Although the results at 2 and 5 years and using the total number of deaths are promising, they should be interpreted with caution because the analyses are subject to a number of possible biases and problems, which are discussed below.

### Discussion

This paper aimed to assess the role of adjuvant chemotherapy in soft-tissue sarcoma, by reviewing all published trials which have randomised patients to receive adjuvant chemotherapy or no adjuvant chemotherapy following surgery (± radiotherapy). Unfortunately, it has not been possible through either a qualitative or quantitative review of the literature to resolve this issue completely satisfactorily.

The qualitative review suffers from the difficulties inherent in trying to review a number of apparently equivocal or conflicting trials and no firm conclusions can be drawn. Previous reviews of the literature (Delaney *et al.*, 1991; Mazanet and Antman, 1991; Elias, 1993; Mertens and Bramwell, 1993; Zalupski, 1993*a*) have been similarly inconclusive.

Despite being more objective and authoritative and producing an encouraging result, the meta-analysis based on data extracted from the literature suffers from a number of possible biases. For example, publication bias, inappropriate patient exclusions, variable follow-up times and a fixed time point analysis can all lead to an overestimate of the size of the treatment effect and its significance compared with that obtained with the individual patient data (Stewart and Parmar, 1993). Furthermore, the principal analyses we have been able to perform were based on estimated 2 year and 5 year survival figures. Such analyses take no account of the relative pattern of survival before 2 years, between 2 and 5 years and after the 5 year time point and, thus, give little indication of the overall survival experience on the two treatments. A more detailed analysis over many different time periods was not possible, as the information required could not be adequately extracted from the trial publications.

Using the overall numbers of deaths in each trial to calculate an OR can also be problematic and was the method used in another meta-analysis of the published results (Jones *et al.*, 1991). Not all trial publications report the overall number of deaths, and the length of follow-up varies considerably among those that do. Therefore, the calculated OR for each trial is based on a different point in time. This may be appropriate if death ratios remain constant over time (the hazards are proportional), but not, for example, if survival curves converge or even diverge after some years. As this may well be the case with chemotherapy in STS, it is not altogether clear how to interpret this type of analysis.

Another similar meta-analysis of the published literature (Zalupski *et al.*, 1993*b*) used an alternative method (logistic regression) to combine overall survival rates, but concentrated on trials including STS of the extremities. Again there was a strong indication of treatment benefit in terms of survival. However, despite trying to control for the estimated sample size and length of follow-up in each trial, other potential sources of bias such as publication bias, patient exclusions, censoring and the use of single time point estimates of survival still exist. Therefore, this result must also be regarded with caution.

Our experience in undertaking this meta-analysis based on published data reflects that of other workers carrying out similar projects in different disease sites. They concluded that it was not possible to perform meta-analyses of the literature

| Reference | No. events/no. entered Chemo | No chemo | O-E | Variance | Odds ratio |
|---|---|---|---|---|---|
| GOG | 29/67 | 39/72 | -3.78 | 8.74 | 0.65 (0.27–1.55) |
| Roswell | 3/8 | 7/11 | -1.21 | 1.22 | 0.37 (0.04–3.83) |
| ECOG | 3/7 | 3/6 | -0.23 | 0.87 | 0.77 (0.05–12.20) |
| SSG | 21/60 | 22/57 | -1.05 | 6.85 | 0.86 (0.32–2.30) |
| Rizzoli | 3/23 | 10/30 | -2.64 | 2.46 | 0.34 (0.07–1.77) |
| MDAH | 5/20 | 9/23 | -1.51 | 2.40 | 0.53 (0.10–2.82) |
| Mayo | 3/26 | 7/27 | -1.91 | 2.07 | 0.40 (0.07–2.39) |
| NCI 1 | 7/39 | 11/28 | -3.48 | 3.25 | 0.34 (0.08–1.44) |
| NCI 2 | 4/12 | 4/10 | -0.36 | 1.32 | 0.76 (0.08–7.16) |
| EORTC | 46/145 | 64/172 | -4.32 | 17.88 | 0.79 (0.43–1.45) |
| Bergonie | 4/26 | 14/23 | -5.55 | 2.90 | 0.15 (0.03–0.67) |
| Total | 128/433 | 190/459 | -26.0 | 49.96 | 0.59 (0.45–0.78) |

**Figure 2** Meta-analysis, at 5 years, of published randomised trials of adjuvant chemotherapy for soft-tissue sarcoma.

to address specific questions in lung (Nicolucci et al., 1989) and ovarian (Marsoni et al., 1990) cancer because of the poor quality of data retrieved from the publications and the subsequent assumptions required to perform the analyses.

In addition, this type of meta-analysis based on published data does not allow the investigation of whether any observed effect is consistent across different subgroups, for example disease sites or histological types. Similarly, the issue of whether polychemotherapy and single-agent chemotherapy are equally effective could not be examined. These additional questions can only be investigated in a meta-analysis of individual patient data. Likewise, by collecting data for each patient, those subsets of patients that received neoadjuvant chemotherapy (Antman et al., 1984; Picci et al., 1988) could be excluded from the analysis.

There may be sufficient evidence from completed randomised trials to assess whether chemotherapy does or does not improve the survival of patients with STS. Unfortunately, it has not been possible to synthesise satisfactorily the results of individual trials on the basis of the information given in the literature in either a qualitative or a quantitative manner. The more objective, but nevertheless flawed, meta-analysis of the published data does provide evidence to suggest that chemotherapy may improve the survival of patients with this disease. However, the only reliable means of confirming this preliminary result is to collect individual data on all patients randomised, in all eligible trials and to combine the results of these trials through an appropriate time-to-event analysis, stratified by trial. We have therefore initiated an international collaborative meta-analysis to collect these data. Both unpublished and published trials will be sought by electronic database searching, hand searching of relevant journals, review article bibliographies, meeting abstracts and trial registers and by contacting experts in the field. Furthermore, using time-to-event data from all patients randomised in intention-to-treat analyses will allow us to explore whether any effect of adjuvant chemotherapy is consistent across disease sites, histological types and grades of disease and with tumour size and the extent of resection.

### References

ALVEGÅRD TA, SIGURDSSON H, MOURIDSEN H, SOLHEIM Ø, UNSGAARD B, RINGBORG U, DAHL O, NORDENTOFT A, BLOMQVIST C, RYDHOLM A, STENER B AND RANSTAM J. (1989). Adjuvant chemotherapy with doxorubicin in high grade soft tissue sarcoma: a randomized trial of the Scandinavian Sarcoma Group. *J. Clin. Oncol.*, **7**, 1504–1513.

ANTMAN K, SUIT H, AMATO D, CORSON J, WOOD W, PROPPE K, HARMON D, CAREY R, GREENBERG J, BLUM R AND WILSON R. (1984). Preliminary results of a randomized trial of adjuvant doxorubicin for sarcomas: lack of apparent difference between treatment groups. *J. Clin. Oncol.*, **2**, 601–608.

BAKER LH, FRANK J, FINE G, BALCERZAK SP, STEPHENS RL, STUCKEY WJ, RIVKIN S, SAIKI J AND WARD JH. (1987). Combination chemotherapy using adriamycin, DTIC, cyclophosphamide, and actinomycin D for advanced soft tissue sarcomas: a randomized comparative trial. A phase III Southwest Oncology Group study (7631). *J. Clin. Oncol.*, **5**, 851–861.

BAKER LH. (1988). Adjuvant therapy for soft tissue sarcomas. In *Recent Concepts in Sarcoma Treatment*, Ryan JR and Baker LH (eds) pp. 130–135. Kluwer Academic Publishers: Dordrecht.

BENJAMIN RS, TERJANIAN TO, FENOGLIO CJ, BARKLEY HT, EVANS HL, MURPHY WK AND MARTIN RG. (1987). The importance of combination chemotherapy for adjuvant treatment of high-risk patients with soft-tissue sarcomas of the extremities. In *Adjuvant Therapy of Cancer*, Vol. V, Salmon SE (ed.) pp. 735–744. Grune & Stratton: Orlando.

BORDEN EC, AMATO DA, EDMONSON JH, RITCH PS AND MASANORI S. (1990). Randomized comparison of doxorubicin and vindesine to doxorubicin for patients with metastatic soft-tissue sarcomas. *Cancer*, **66**, 862–867.

BRAMWELL V, ROUESSE J, STEWARD W, SANTORO A, SCHRAFFORDT-KOOPS H, BUESA J, RUKA W, PRIARIO J, WAGENER T, BURGERS M, VAN UNNIK J, CONTESSO G, THOMAS D, VAN GLABBEKKE M, MARKHAM D AND PINEDO H. (1994). Adjuvant CYVADIC chemotherapy for adult soft tissue sarcoma – reduced local recurrence but no improvement in survival: a study of the European Organization for Research and Treatment of Cancer Soft Tissue and Bone Sarcoma Group. *J. Clin. Oncol.*, **12**, 1137–1149.

CHANG AE, KINSELLA T, GLATSTEIN E, BAKER AR, SINDELAR WF, LOTZE MT, DANFORTH DN, SUGARBAKER P, LACK E, STEINBERG SM, WHITE DE AND ROSENBERG SA. (1988). Adjuvant chemotherapy for patients with high-grade soft-tissue sarcomas of the extremity. *J. Clin. Oncol.*, **6**, 1491–1500.

DELANEY TF, YANG JC AND GLATSTEIN E. (1991). Adjuvant therapy for adult patients with soft tissue sarcomas. *Oncology*, **5**, 105–118.

EARLY BREAST CANCER TRIALISTS' COLLABORATIVE GROUP. (1990). *Treatment of Early Breast Cancer*, Vol I. *Worldwide Evidence 1990*. Oxford University Press: Oxford.

EDMONSON JH. (1985). Systemic chemotherapy following complete excision of nonosseous sarcomas: Mayo Clinic experience. *Cancer Treat. Symp.*, **3**, 89–97.

EDMONSON JH, FLEMING TR, IVINS JC, BURGERT EO, SOULE EH, O'CONNELL MJ, SIM FH AND AHMANN DL. (1984). Randomized study of systemic chemotherapy following complete excision of nonosseous sarcomas. *J. Clin. Oncol.*, **2**, 1390–1396.

EILBER FR, GIULIANO AE, HUTH JF AND MORTON DL. (1988). A randomized prospective trial using postoperative adjuvant chemotherapy (Adriamycin) in high-grade extremity soft tissue sarcoma. *Am. J. Clin. Oncol.*, **11**, 39–45.

ELIAS AJ. (1993). Chemotherapy for soft-tissue sarcomas. *Clin. Orthopaed. Rel. Res.*, **289**, 94–105.

GLENN J, KINSELLA T, GLATSTEIN E, TEPPER J, BAKER A, SUGARBAKER P, SINDELAR W, ROTH J, BRENNAN M, COSTA J, SEIPP C, WESLEY R, YOUNG RC AND ROSENBERG SA. (1985*a*). A randomized, prospective trial of adjuvant chemotherapy in adults with soft tissue sarcomas of the head and neck, breast, and trunk. *Cancer*, **55**, 1206–1214.

GLENN J, SINDELAR W, KINSELLA T, GLATSTEIN E, TEPPER J, COSTA J, BAKER A, SUGARBAKER P, BRENNAN M, SEIPP C, WESLEY R, YOUNG R AND ROSENBERG SA. (1985*b*). Results of multimodality therapy of resectable soft-tissue sarcomas of the retroperitoneum. *Surgery*, **97**, 316–325.

JONES GW, CHOUINARD E AND PATEL M. (1991). Adjuvant Adriamycin (doxorubicin) in adult patients with soft-tissue sarcomas: a systematic overview and quantitative meta-analysis. *Clin. Invest. Med.*, **14** (Suppl. 19), A772.

KINSELLA T, SINDELAR W, LACK E, GLATSTEIN E AND ROSENBERG SA. (1988). Preliminary results of a randomized study of adjuvant radiation therapy in resectable adult retroperitoneal soft tissue sarcomas. *J. Clin. Oncol.*, **6**, 18–25.

LERNER HJ, AMATO DA, SAVLOV E, DEWYS WD, MITTELMAN A, URTASUN RC, SOBEL S AND SHIRAKI M. (1987). Eastern Cooperative Oncology Group: a comparison of adjuvant doxorubicin and observation for patients with localized soft tissue sarcoma. *J. Clin. Oncol.*, **5**, 613–617.

MAZANET R AND ANTMAN KH. (1991). Adjuvant therapy for sarcomas. *Semin. Oncol.*, **18**, 603–612.

MARSONI S, TORRI V, TAIANA A, GAMBINO A, GRILLI R, LIATI P, FRANZOSI MG, PISTOTTI V, FOCARILE F AND LIBERATI A. (1990). Critical review of quality and development of randomised clinical trials (RCTs) and their influence on the treatment of advanced epithelial ovarian cancer. *Ann. Oncol.*, **1**, 343–350.

MERTENS WC AND BRAMWELL VHC. (1993). Adjuvant chemotherapy in the treatment of soft-tissue sarcoma. *Clin. Orthopaed. Rel. Res.*, **289**, 81–93.

NICOLUCCI A, GRILLI R, ALEXANIAN A, APOLONE G, TORRI V AND LIBERATI A. (1989). Quality evaluation and clinical implications of randomised controlled clinical trials on the treatment of lung cancer: lost opportunity for meta-analysis. *JAMA*, **262**, 2101–2107.

OMURA A, BLESSING JA, MAJOR F, LIFSHITZ S, EHRLICH CE, MANGAN C, BEECHAM J, PARK R AND SILVERBERG S. (1985). A randomised trial of adjuvant adriamycin in uterine sarcomas: a Gynecologic Oncology Group study. *J. Clin. Oncol.*, **3**, 1240-1245.

PICCI P, BACCI G, GHERLINZONI F, CAPANNA R, MERCURI M, RUGGIERI P, BALDINI N, AVELLA M, PIGNATTI G AND MANFRINI M. (1988). Results of randomized trial for the treatment of localized soft tissue tumors (STS) of the extremities in adult patients. In *Recent Concepts in Sarcoma Treatment*, Ryan JR and Baker LH (eds) pp. 144-148. Kluwer Academic Publishers: Dordrecht.

PINEDO HM, BRAMWELL VHC, MOURIDSEN HT, SOMERS R, VENDRIK CPJ, SANTORO A, BUESA J, WAGENER TH, VAN OOSTEROM AT, VAN UNNIK JAM, SYLVESTER R, DE PAUW M, THOMAS D AND BONADONNA G. (1984). Cyvadic in advanced soft tissue sarcoma: a randomized study comparing two schedules. *Cancer*, **53**, 1825-1832.

PINEDO HM AND VERWEIJ J. (1986). The treatment of soft tissue sarcomas with focus on chemotherapy: a review. *Radiother. Oncol.*, **5**, 193-205.

PIVER MS, SHASHIKANT BL, MARCHETTI DL AND EMRICH LJ. (1988). Effect of adjuvant chemotherapy on time to recurrence and survival of stage I uterine sarcomas. *J. Surg. Oncol.*, **38**, 233-239.

RAVAUD A, BUI NB, COINDRE JM, KANTOR G, STOCKLE E, LAGARDE Y, BECOUARN Y, CHAUVERGNE J, BONICHON F AND MAREE D. (1990). Adjuvant chemotherapy with Cyvadic in high risk soft tissue sarcoma: a randomized prospective trial. In *Adjuvant Therapy of Cancer*, Vol. VI, Salmon SE (ed.) pp. 556-566. WB Saunders: Philadelphia.

ROSENBERG SA, TEPPER J, GLATSTEIN E, COSTA J, YOUNG R, BAKER A, BRENNAN MF, DEMOSS EV, SEIPP C, SINDELAR WF, SUGARBACKER P AND WESLEY R. (1983). Prospective randomized evaluation of adjuvant chemotherapy in adults with soft tissue sarcomas of the extremities. *Cancer*, **52**, 424-434.

ROSENBERG SA, CHANG AE AND GLATSTEIN E. (1985). Adjuvant chemotherapy for treatment of extremity soft tissue sarcomas: review of National Cancer Institute experience. *Cancer Treat. Symp.*, **3**, 83-88.

SCHOENFELD DA, ROSENBAUM C, HORTON J, WOLTER JM, FALKSON G AND DECONTI RC. (1982). A comparison of adriamycin versus vincristine and adriamycin, and cyclophosphamide versus vincristine, actinomycin-D, and cyclophosphamide for advanced sarcoma. *Cancer*, **50**, 2757-2762.

SOUHAMI R AND TOBIAS J. (1986). Bone and soft tissue sarcoma. In *Cancer and its Management*, pp. 389-405. Blackwell Scientific Publications: Oxford.

STEWART LA. (1992). The role of overviews. In *Introducing New Treatments for Cancer: Practical, Ethical and Legal problems*, Williams CJ (ed.) pp. 384-401. John Wiley: Chichester.

STEWART LA AND PARMAR MKB. (1993). Meta-analysis of the literature or of individual patient data: is there a difference? *Lancet*, **341**, 418-422.

ZALUPSKI MM, RYAN JR, HUSSEIN ME AND BAKER LH. (1993*a*). Systemic adjuvant chemotherapy for soft tissue sarcomas of the extremities. *Surg. Oncol. Clins. N. Am.*, **2**, 621-637.

ZALUPSKI MM, RYAN JR, HUSSEIN ME AND BAKER LH. (1993*b*). Defining the role of adjuvant chemotherapy for patients with soft tissue sarcoma of the extremities. In *Adjuvant Therapy of Cancer*, Vol. VII, Salmon SE (ed.) pp. 385-392. Lippincott: Philadelphia.